# Association of microRNA-126 expression with clinicopathological features and the risk of biochemical recurrence in prostate cancer patients undergoing radical prostatectomy

**DOI:** 10.1186/1746-1596-8-208

**Published:** 2013-12-18

**Authors:** Xiaoke Sun, Zhe Liu, Zhen Yang, Lin Xiao, Feng Wang, Yang He, Pengxiao Su, Junhu Wang, Baoli Jing

**Affiliations:** 1 Department of Surgery, Xi’an Hong Hui Hospital, Xi’an 710054, China; 2 Department of Urology, Xi’an Electricity Power Center Hospital, State Grid Corporation of China, Xi’an 710032, China

**Keywords:** Prostate cancer, MicroRNA-126, Clinical pathology, Prognosis

## Abstract

**Objective:**

Numerous studies have suggested that microRNA-126 (miR-126) is involved in development of various cancer types as well as in malignant proliferation and invasion. However, its role in human prostate cancer (PCa) is still unclear. The aim of this study was to investigate miR-126 expression in PCa and its prognostic value for PCa patients undergoing radical prostatectomy.

**Methods:**

A series of 128 cases with PCa were evaluated for the expression levels of miR-126 by quantitative reverse-transcription PCR (qRT-PCR). Kaplan-Meier analysis and Cox proportional hazards regression models were used to investigate the correlation between miR-126 expression and prognosis of PCa patients.

**Results:**

Compared with non-cancerous prostate tissues, the expression level of miR-126 was significantly decreased in PCa tissues (PCa vs. non-cancerous prostate: 1.05 ± 0.63 vs. 2.92 ± 0.98, P < 0.001). Additionally, the loss of miR-126 expression was dramatically associated with aggressive clinical pathological features, including advanced pathological stage (P = 0.001), positive lymph node metastasis (P = 0.006), high preoperative PSA (P = 0.003) and positive angiolymphatic invasion (P = 0.001). Moreover, Kaplan–Meier survival analysis showed that PCa patients with low miR-126 expression have shorter biochemical recurrence (BCR)-free survival than those with high miR-126 expression. Furthermore, multivariate analysis indicated that miR-126 expression was an independent prognostic factor for BCR-free survival after radical prostatectomy.

**Conclusion:**

These findings suggest for the first time that the loss of miR-126 expression may play a positive role in the malignant progression of PCa. More importantly, the downregulation of miR-126 may serve as an independent predictor of BCR-free survival in patients with PCa.

**Virtual slides:**

The virtual slide(s) for this article can be found here: http://www.diagnosticpathology.diagnomx.eu/vs/1740080792113255.

## Introduction

Prostate cancer (PCa) represents one of the most prevalent malignancies among men worldwide and the second most frequent cause of male cancer-related death in most Western countries [1]. PCa is a clinically heterogeneous-multifocal and highly aggressive disease [2]. Even when the tumor is apparently confined to prostate, it encompasses a broad spectrum of diseases, some of which are characterized by extremely indolent behavior and others by very poor outcome [3]. Despite the advancement of therapeutic strategies, there are few effective therapeutic options for advanced PCa. The annual morbidity rate of this cancer has increased by 14% since 1990 [4]. It is the most troublesome aspect that how to diagnose PCa patients at early stage. Several clinicopathological features, including tumor volume, pathological grade, status of lymph node metastasis, preoperative PSA, Gleason score, have been used for diagnosis and prognosis [5,6]. However, there is no widely accepted method for quantifying tumor volume [7]; pathological grade scoring methods can result in significant inter-observer variations, particularly when defining intermediate tumor grades [8]; accumulating studies have found that PCa patients with the equivalent PSA level could have various clinical outcomes because of the molecularly heterogeneous subtypes [9]. In order to overcome these limitations, efficient diagnostic and prognostic biomarkers for PCa are extremely necessary for the early detection of aggressive PCa and for the improvement of clinical outcome of patients with this disease.

MicroRNAs (miRNAs), which are small non-coding RNA molecules with 19–24 nucleotides in length, play crucial roles in the regulation of basic biological processes, including cell growth, apoptosis and differentiation, by silencing specific target genes through impairing their translation or both specifically binding to the 3′-untranslated regions (3′UTR) of target mRNAs [10]. Until now, there have been more than 1,400 human miRNA sequences identified and many of them have been demonstrated to be implicated into cancer pathogenesis [11]. Functionally, miRNAs can act either as oncogenes or tumor suppressors according to the roles of their target genes. With regard to the relationship between cancer and miRNAs, accumulating studies have detected the specific miRNA profiles in various solid tumors and several miRNA signatures have been identified as diagnostic markers or therapeutic targets. Especially in PCa, Walter et al. [12] performed a comprehensive differential miRNA expression analysis of a group of PCa patients with different Gleason score compared to their corresponding normal epithelium and stromal tissue. Mavridis et al. [13] comprehensively profiled the expression of the mature miRNA-224 molecule in benign prostatic hyperplasia and PCa with a reliable and cost-efficient method based on quantitative real-time PCR. They also indicated that miR-224 may be downregulated in PCa, that miR-244 expression may be gradually decreased as malignancy progresses, and that miR-224 expression may be associated with favorable prognosis. These findings suggest that miRNAs have a relevant role as biomarkers in PCa, and it is of great significance to screen miRNA markers with diagnostic and prognostic values for patients with PCa.

MicroRNA-126 (miR-126) originates from a common precursor structure located within intron 7 of epidermal growth factor-like domain 7 (EGFL7) [14]. It is highly expressed in vascular endothelial cells and functions as a key positive regulator to promote angiogenesis in response to angiogenic growth factors by repressing negative regulators of signal transduction pathways [15]. Recent studies have found the involvement of miR-126 in various human malignancies. For example, miR-126 may be downregulated in non-small cell lung tumor tissues, and may correlate with microvessel density and clinical outcomes [16]; the decreased expression of miR-126 may be associated with poor metastasis-free survival of breast cancer patients [17]; Loss of miR-126 may be more frequent in colorectal cancers with metastasis [18]. In contrast, miR-126 may be act as oncogene since it has been demonstrated to be a significant negative prognostic factor for squamous cell carcinomas [19]. These contradictory results may indicate that the roles of miR-126 in various tissues may be different because of its high tissue specificity. Notably, Saito et al. [20] in 2009 showed that miR-126 was downregulated in PCa cell lines. Walter et al. [12] in 2013 by PCR array profiling identified miR-126 as one of the downregulation miRNAs in high grade PCa. However, little is known about the expression of miR-126 in human PCa tissues, and data on its potential prognostic value in PCa are completely lacking.

In the current study, a series of 128 cases with PCa were evaluated for the expression levels of miR-126 by quantitative reverse-transcription PCR (qRT-PCR). We investigated the correlation between the relative expression of miR-126 and clinicopathological parameters to evaluate its clinical significance. Additionally, we assessed the influence of miR-126 expression on the biochemical recurrence (BCR) of PCa patients.

## Materials and methods

### Patients and tissue samples

The study was approved by the Research Ethics Committee of Xi’an hong hui hospital and Xi’an electricity power Center Hospital. Written informed consent was obtained from all of the patients. All specimens were handled and made anonymous according to the ethical and legal standards.

One hundred and twenty-eight primary PCa and corresponding noncancerous prostate tissue samples from the same specimens (n = 128) were collected from Xi’an hong hui hospital and Xi’an electricity power Center Hospital, from 1996 to 2008. None of the patients received androgen deprivation treatment, chemotherapy, or radiation therapy prior to radical prostatectomy. All 128 patients with PCa received radical prostatectomy. The complete records of the cases pre- and post-operation, and samples of the primary tissue, had been preserved. The following clinicopathological parameters, including preoperative PSA, Gleason score, pathological stage, lymph node status, angiolymphatic invasion, margin status, and biochemical relapse, were recorded. The clinicopathological information of the patients is shown in Table 1.

**Table 1:** Correlation of miR-126 expression with clinicopathological features of PCa patients

**Clinicopathological features**	**Cases No (n, %)**	**miR-126 expression status**		**P**
		**Low (n, %)**	**High (n, %)**	
**Age**				
<70	70 (54.69)	39 (55.71)	31 (44.29)	NS
≥70	58 (45.31)	29 (50.00)	29 (50.00)	
**Preoperative PSA**				
<4 ng/mL	3 (2.34)	0 (0)	3 (100.00)	0.003
4-10 ng/mL	35 (27.34)	7 (20.00)	28 (80.00)	
>10 ng/mL	90 (70.31)	61 (67.77)	29 (32.23)	
**Gleason score**				
4-6	62 (48.44)	31 (50.00)	31 (50.00)	NS
7	30 (23.44)	19 (63.33)	11 (36.67)	
8-10	36 (28.13)	18 (50.00)	18 (50.00)	
**Pathological stage**				
T1	72 (56.25)	22 (30.56)	50 (69.44)	0.001
T2/T3	56 (43.75)	46 (82.14)	10 (17.86)	
**Lymph node metastasis**				
Negative	106 (82.81)	47 (44.34)	59 (55.66)	0.006
Positive	22 (17.19)	21 (95.45)	1 (4.55)	
**Angiolymphatic invasion**				
Negative	110 (85.94)	50 (45.45)	60 (54.55)	0.001
Positive	18 (14.06)	18 (100.00)	0 (0)	
**Surgical margin status**				
Negative	108 (84.38)	56 (51.85)	52 (48.15)	NS
Positive	20 (15.62)	12 (60.00)	8 (40.00)	

Note: ‘NS’ refers to the difference without statistic significance.

All 128 patients with PCa were given a follow-up exam ranging from three to ten years. All the patients who died from diseases other than PCa or from unexpected events were excluded from the case collection. For the analysis of biochemical recurrence-free survival, the date of prostatectomy was used to represent the beginning of the follow-up period. The endpoint was the time to biochemical relapse which was defined as the period between surgical treatment and the measurement of two successive values of serum PSA level ≥ 0.2 ng/ml.

### qRT-PCR

RNA was extracted from fresh tissues of patients with PCa using Trizol Reagent (Invitrogen, USA) according to the manufacturer’s protocol. One microgram of total RNA was reverse-transcribed using SuperScrip III (Invitrogen, USA). After cDNA was synthesized with a miRNA-specific stem-loop primer, the quantitative PCR was performed with the specific primers as follows: miR-126_F 5′-GTCGTATCCAGTGCAGGGTCCGAG-3′; miR-126_R, 5′-GTATTCGCACTGGATACGAC-3′; U6_F, 5′-CTCGCTTCGGCAGCACA-3′; U6_R, 5′-AACGCTTCACGAATTTGCGT-3′. Real-time PCR was performed using an Applied Biosystems 7500 real-time PCR system using 1 μl reverse transcriptase samples in a 20 μl final reaction mixture. 1X TaqMan Universal PCR master mix (Takara, Japan) was used for general PCR. RNU6B was used as an internal control. Relative quantification of target miRNA expression was evaluated using the comparative cycle threshold (CT) method. Each sample was examined in triplicate and the amounts of the PCR products produced were nonneoplasticized to RNU6B.

### Statistical analysis

Statistical analysis was performed using the software of SPSS version12.0 for Windows (SPSS Inc, IL, USA). Continuous variables were expressed as mean ± S.D. Fisher’s exact test and Pearson *χ*^2^ test were respectively used to analyze 2 × 2 tables and non-2 × 2 tables. Kaplan-Meier and Cox Regression methods were used for the question of survival analysis. Differences were considered statistically significant when *p* was less than 0.05.

## Results

### Downregulation of miR-126 in PCa tissues

**Figure 1:**
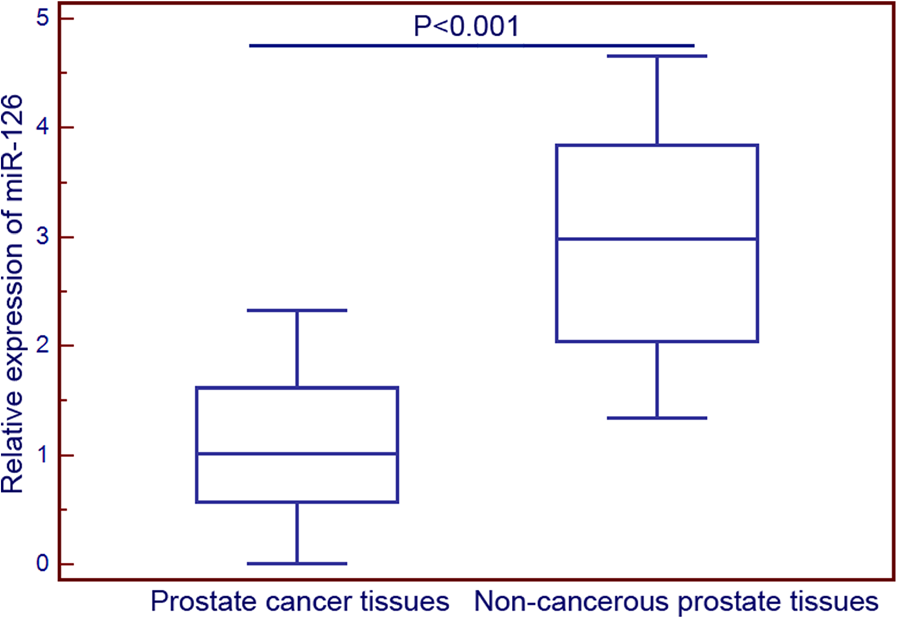
**microRNA-126 (miR-126) expression in 128 prostate cancer (PCa) tissues and matched non-cancerous prostate tissues normalized to RNU6B detected by quantitative reverse-transcription PCR (qRT-PCR) assay.** The expression levels of miR-126 were detected and analyzed in 128 pairs of PCa and adjacent non-cancerous prostate tissues by qRT-PCR analysis. The results showed that miR-126 expression level was significantly lower in PCa tissues compared to that in adjacent non-cancerous prostate tissues (1.05 ± 0.63 vs. 2.92 ± 0.98, P < 0.001).

The expression levels of miR-126 were detected and analyzed in 128 pairs of PCa and adjacent non-cancerous prostate tissues by qRT-PCR analysis. The results showed that miR-126 expression level was significantly lower in PCa tissues compared to that in adjacent non-cancerous prostate tissues (1.05 ± 0.63 vs. 2.92 ± 0.98, P < 0.001, Figure 1). Moreover, the median of miR-126 expression levels in all PCa tissues was 1.02. Thus, the miR-126 expression levels were further analyzed by classifying as low (n = 68, based on a relative expression level less than 1.02) and as high (n = 60, based on a relative expression level greater than 1.02).

### Downregulation of miR-126 correlates with aggressive clinicopathological features of patients with PCa

Table 1 summarized the correlation between miR-126 expression and clinicopathological features of patients with PCa. Our data showed that low miR-126 expression was significantly correlated with aggressive clinical pathological features, including advanced pathological stage (P = 0.001), positive lymph node metastasis (P = 0.006), high preoperative PSA (P = 0.003) and positive angiolymphatic invasion (P = 0.001). However, the expression status of miR-126 was not associated with patients’ age, Gleason score, and surgical margin status.

### Prognostic value of miR-126 expression in PCa patients

**Figure 2:**
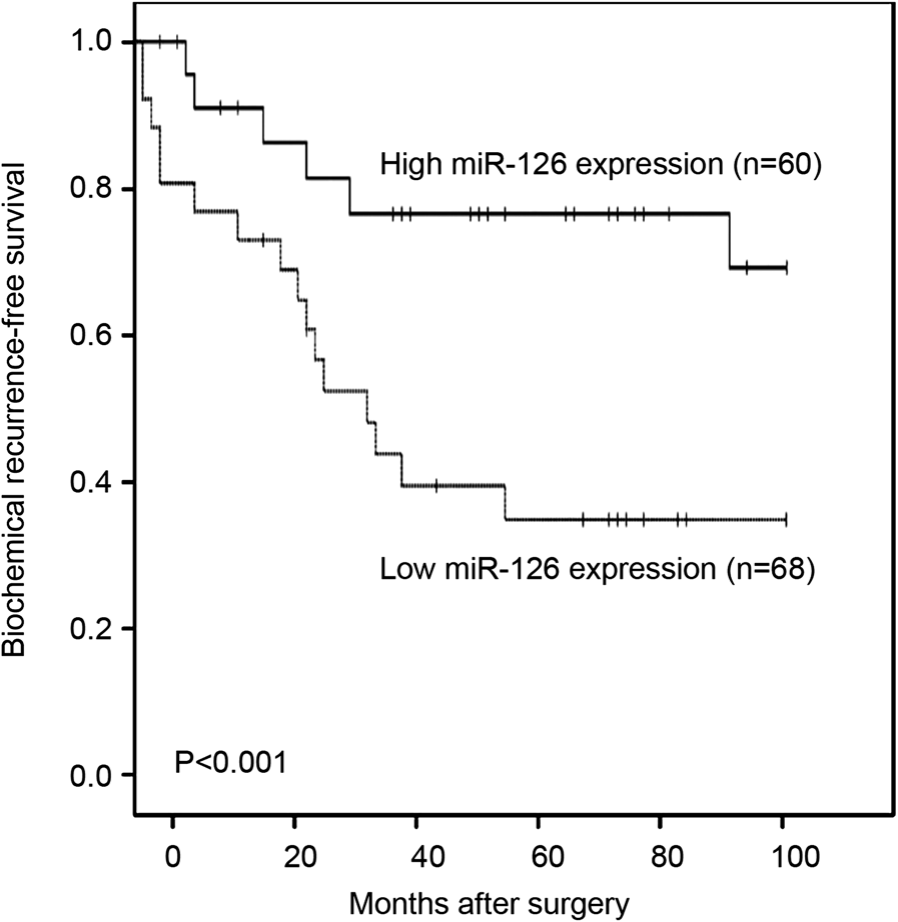
**Biochemical recurrence (BCR)-free survival curves for two groups defined by low and high expression of miR-126 in patients with PCa.** The patients with low miR-126 expression had significantly shorter BCR-free survival after radical prostatectomy than patients with high miR-126 expression did (P < 0.001).

To evaluate the possible prognostic value of miR-126, we performed BCR-free survival analysis for all 128 PCa patients undergoing radical prostatectomy. As shown in Figure 2, Kaplan-Meier curves were plotted between high or low miR-126 expression and BCR-free survival. Notably, patients with low miR-126 expression had significantly shorter BCR-free survival after radical prostatectomy than patients with high miR-126 expression did (P < 0.001; Figure 2). In addition, the univariate analysis with Cox proportional hazards model found that miR-126 expression status (P < 0.001), pathological stage (P < 0.001), lymph node metastasis status (P = 0.002), and positive angiolymphatic invasion (P < 0.001) were significantly associated with BCR-free survival, while patients’ age, preoperative PSA, Gleason score, and surgical margin status were not significant factors (all P > 0.05, Table 2).

**Table 2:** Univariate survival analysis of biochemical recurrence (BCR)-free survival in 128 patients with PCa

**Variables**	**BCR-free survival**		
	**Exp (B)**	**95% CI**	**P**
**Age (<70 vs. ≥70)**	2.68	0.17–5.03	NS
**Preoperative PSA (<10 ng/mL vs. ≥10 ng/mL)**	2.34	0.11–4.48	NS
**Gleason score (4–6 vs. 7–10)**	2.26	0.10–4.32	NS
**Pathological stage (T1-2 vs. T3-4)**	5.61	1.12–11.28	<0.001
**Lymph node metastasis (Negative vs. Positive)**	4.13	0.82–8.22	0.002
**Angiolymphatic invasion (Negative vs. Positive)**	5.62	1.12–11.39	<0.001
**Surgical margin status (Negative vs. Positive)**	2.27	0.10–4.82	NS
**miR-126 expression (High vs. Low)**	5.68	1.19–11.33	<0.001

Note: ‘NS’ refers to the difference without statistic significance.

Furthermore, the Cox multivariate analysis demonstrated the value of miR-126 expression, and other clinicopathologic features for predicting BCR-free survival of patients with PCa. As shown in Table 3, miR-126 expression status (P = 0.01), pathological stage (P = 0.001), lymph node metastasis status (P = 0.03), and positive angiolymphatic invasion (P = 0.001) were all independent prognostic factors for predicting BCR-free survival of patients with PCa.

**Table 3:** Multivariate survival analysis of biochemical recurrence (BCR)-free survival in 128 patients with PCa

**Variables**	**BCR-free survival**		
	**Exp (B)**	**95% CI**	**P**
**Pathological stage**	4.11	1.02–8.08	0.001
**Lymph node metastasis**	3.13	0.81–4.20	0.03
**Angiolymphatic invasion**	4.62	1.13–9.09	0.001
**miR-126 expression**	3.68	0.99–6.83	0.01

Note: ‘NS’ refers to the difference without statistic significance.

## Discussion

Although patients with localized PCa receiving radical prostatectomy have experienced long-term survival, the BCR, which is generally considered as the earliest indicator of recurrent disease, occurs in nearly half of patients following surgery on long-term follow-up [21]. Thus, it is very important to detect the early risk of BCR in order to improve the prognosis of patients with PCa. Hua et al. [22] identified GOLPH3 as an important prognostic factor for patients with PCa; Jonsson et al. [23] demonstrated that high RBM3 expression in PCa may independently predict a reduced risk of BCR and disease progression. In this study, we detected the aberrant expression of miR-126 in a large PCa cohort. As the results, we firstly found that the expression level of miR-126 in PCa tissues was significantly lower than that in adjacent non-cancerous prostate tissues. In addition, the downregulation of miR-126 was significantly associated with aggressive clinicopathological features, including advanced pathological stage, positive lymph node metastasis, high preoperative PSA and positive angiolymphatic invasion, but not with patients’ age, Gleason score, and surgical margin status. Moreover, we proved that miR-126 expression was significantly associated with BCR-free survival of patients with PCa. More importantly, the multivariate analyses showed that loss of miR-126 expression was an independent predictor of shorter BCR-free survival in patients with PCa. These findings suggest that miR-126 may play crucial roles in the pathogenesis and aggressiveness of PCa, and miR-126 downregulation especially may be correlated with the unfavorable prognosis in PCa. To our knowledge, this is the first study to investigate the association between miR-126 and PCa.

miR-126, an endothelial-specific miRNA, is generated by EGFL7 pre-mRNA splicing [24]. Its expression is regulated by of EGFL7 gene promoter [24]. Since an intronic miRNA tends to be co-expressed with its host gene, miR-126 and its host gene, EGFL7, are both downregulated by DNA methylation, with restoration of expression levels by epigenetic treatment [25]. Functionally, miR-126 governs angiogenesis and vascular integrity [26]. miR-126 downregulation in the endothelial cells leads to the activation of vascular cell adhesion molecule-1 and subsequently triggers adhesion of leukocytes to the injured vessels to induce inflammatory changes. During atherosclerosis-induced apoptosis in the endothelium, miR-126 is released into the apoptotic bodies and confers a counteraction of apoptosis and recruitment of vascular progenitor cells to the site of injury [27,28]. Since these processes are implicated into the developing cancer stroma, miR-126 may play a role in carcinogenesis and cancer progression. A number of studies have demonstrated that miR-126 acts as a tumor suppressor gene in various human cancers. The downregulation of miR-126 has been observed in adult T-cell leukemia [29], oral squamous cell carcinoma [19,30], nonsmall cell lung cancer [16], breast cancer [17], cervical cancer [31], hepatocellular carcinoma [32], colorectal cancer [18], and gastric cancer [33]. In line with these previous studies, our data here also showed the loss of miR-126 expression in human PCa tissues, suggesting that the aberrant expression of miR-126 may be involved in the carcinogenesis of prostate. Regarding the clinical significance of miR-126, accumulating studies have indicated that the downregulation of miR-126 may be associated with advanced tumor progression and unfavorable outcome in cancer patients, which is also similar with our results in the current study. Therefore, it is conceivable that miR-126 may play an important role in tumor metastasis and tumor recurrence of PCa.

The limitation of the current study is that we did not investigate the mechanism of miR-126 acting on PCa. In breast cancer, the role of miR-126 in tumor metastasis may be related to the negative regulation of insulin receptor substrate-1 expression and it inhibits endothelial cell recruitment and angiogenesis through the negative regulation of IGFBP2/IGF1/IGF1R and GAS6/ MERTK signaling pathways [17]. In non-small cell lung cancer, miR-126 blocked the activity of Crk leading to suppressing the tumor cell metastasis [16]. In gastric cancer tissues, miR-126 can also directly inhibit VEGF signaling pathways, including expression of PIK3R2 and p85-b, thereby playing a role in anti-angiogenesis and inhibition of vascular integrity [33]. In colon cancer, miR-126 suppresses cancer cell proliferation and invasion via inhibiting RhoA/ROCK signaling pathway [18]. In cervical cancer, the repression of miR-126 may facilitate tumor angiogenesis and invasion growth by upregulating a proangiogenic gene adrenomedullin [31]. However, the precise molecular mechanisms behind the aberrant expression of miR-126 in PCa are still unclear. Additional studies to address this problem may be essential and required.

In conclusion, our data suggest for the first time that loss of miR-126 may play a positive role in the malignant progression of PCa. More importantly, the downregulation of miR-126 may serve as an independent predictor of BCR-free survival of patients with PCa.

## Competing interests

The authors report no conflicts of interest. The authors alone are responsible for the content and writing of the paper.

## Authors’ contribution

XKS designed this study. XKS, ZL and ZY performed the experiments and drafted the manuscript. LX, FW, YH, PXS, JHW, BLJ participated in sample collection. All authors read and approved the final manuscript.
